# Exploring Vaginal Birth After Cesarean: Success Rates and Cultural Decisions in the United Arab Emirates

**DOI:** 10.7759/cureus.54880

**Published:** 2024-02-25

**Authors:** Shazia Tariq, Nilanjana Singh

**Affiliations:** 1 Obstetrics and Gynecology, Kanad Hospital, Abu Dhabi, ARE

**Keywords:** united arab emirates (uae), cultural decision, success rate, neonatal intensive care unit (nicu), vbac

## Abstract

Objective: This study explores the success rates and cultural influences on the decision-making process for vaginal birth after cesarean (VBAC) in the United Arab Emirates (UAE).

Methodology: An observational cohort study was conducted at a hospital in the UAE accredited by Joint Commission International, enrolling 263 women eligible for VBAC from March 1, 2018, to February 28, 2019. The study focused on maternal-fetal outcomes, the proportions of women opting for trial of labor after cesarean (TOLAC) versus elective repeat cesarean section (ERCS), and the impact of cultural backgrounds on these decisions.

Results: The study found significant cultural variations in VBAC acceptance and success rates. Among local Emirati/Omani women, 86% (152 out of 177) opted for TOLAC, with an 83% success rate (126 out of 152). In contrast, lower TOLAC uptake and success rates were observed among other nationalities, such as Egyptian and other Arab women. The study also noted higher VBAC success rates in women with prior vaginal deliveries and those who experienced spontaneous labor. NICU admissions and maternal readmissions were lower in the TOLAC group (1% NICU admissions and 2% maternal readmissions) compared to the ERCS group (8.2% NICU admissions).

Conclusion: The study underscores the influence of cultural factors in VBAC decision-making and outcomes, highlighting the need for culturally tailored counseling and care. It also confirms the safety and efficacy of VBAC in appropriately selected cases, advocating for more research into counseling practices and long-term outcomes in culturally diverse populations.

Impact statement: This research adds to the understanding of how cultural and ethnic backgrounds influence VBAC decisions and outcomes, offering critical insights for clinical practice, especially in multicultural societies like the UAE. It emphasizes the role of tailored counseling and suggests avenues for future research in this domain.

## Introduction

Context and rationale

The landscape of obstetric care has seen a paradigm shift over the past decades with the cesarean delivery rate escalating globally. In the United States, this figure has soared from a modest 5% in 1970 to a substantial 31.9% by 2016, a trend mirrored across various healthcare systems [1]. Simultaneously, vaginal birth after cesarean (VBAC) has presented as a clinically viable option, though its uptake fluctuates amidst concerns such as uterine rupture and medico-legal implications [2].

Cultural considerations

In the diverse landscape of the United Arab Emirates (UAE), cultural inclinations profoundly influence childbirth choices. Our hospital serves a predominantly Arab Emirati and Omani female population, with a significant contingent of expatriates from varied nationalities. Cultural predilections are observed, wherein local Arab women exhibit a pronounced preference for vaginal delivery [3]. However, the acceptance rates for the trial of labor after cesarean (TOLAC) and the consequent VBAC success vary noticeably between different cultural groups, presenting a unique opportunity to examine the interplay between cultural background and birthing choices.

Research gap and study focus

While existing research has extensively explored demographic factors influencing cesarean section rates, there remains a paucity of data on how cultural nuances within a country like the UAE affect these decisions [3,4]. To address this gap, our study will scrutinize maternal-fetal outcomes, the decision-making process for TOLAC versus elective repeat cesarean section (ERCS), and the subsequent success rates of VBAC, particularly focusing on various nationalities and cultural groups. This approach aligns with the recommendation for more robust predictive models for VBAC, which consider not only clinical indicators such as previous vaginal deliveries and induction methods but also cultural factors [5].

Objectives

The objectives of this study are multifaceted: to evaluate maternal-fetal outcomes in patients eligible for VBAC over the course of one year at our institution; to ascertain the proportions of women who elect for ERCS in comparison to those who undertake TOLAC; to quantify neonatal intensive care unit admissions and maternal readmission rates in these cohorts; and to delineate the cultural underpinnings guiding these birth choices. Moreover, we aim to measure the success rates of VBAC in relation to induction methods, the occurrence of spontaneous labor, and the impact of previous vaginal deliveries on the likelihood of VBAC success.

## Materials and methods

Study design and setting

This observational cohort study was conducted at an institution in the United Arab Emirates accredited by Joint Commission International. The hospital's VBAC clinical care program, certified by the Joint Commission International, serves a majority local population of Arab Emirati and Omani women, alongside a diverse expatriate community. This study was reviewed and approved by the Kanad Hospital Research Ethics Board, Abu Dhabi, UAE (approval KND-2018-VBAC).

Participants

All women who attended the hospital and were eligible for vaginal birth after cesarean (previous one lower segment transverse Cesarean) were enrolled in the study from March 1, 2018, to February 28, 2019. Exclusion criteria included multiple gestations, gestations less than 36 weeks, previous classical or T-shaped uterine incisions, placenta previa, and extensive prior uterine surgery.

Data collection and consent

Prospective data were collected from electronic medical records, capturing demographics, obstetric history, current pregnancy and labor details, and postnatal outcomes. All participants received detailed antenatal counseling about the risks and benefits of VBAC, supplemented with written information. Informed consent for TOLAC or ERCS was documented after 35 weeks of gestation, following a comprehensive discussion with the patient, ensuring respect for their informed choice.

Interventions and monitoring

Patients needing induction received either a cervical ripening balloon for an unfavorable Bishop’s score (i.e., scores less than 6) or intravenous oxytocin for a favorable cervix. Continuous electronic fetal heart rate monitoring was employed for all patients undergoing TOLAC.

Outcome measures and data recording

The primary outcome measure was the VBAC success rate, with secondary outcomes including NICU admissions and maternal readmissions within six weeks post-delivery. All outcomes were meticulously recorded and subjected to a thorough review process. For statistical analysis of the data, the chi-square test was employed to evaluate the significance of observed differences in VBAC success rates among different nationality groups.

Statistical analysis

We conducted the statistical analysis using Minitab software version 18 (Minitab, LLC, State College, PA, USA). A chi-square test was applied to determine the significance of variations in VBAC success rates among different nationalities. This approach helped assess whether the observed differences were statistically significant, effectively comparing proportions across categorical variables.

## Results

**Figure 1:**
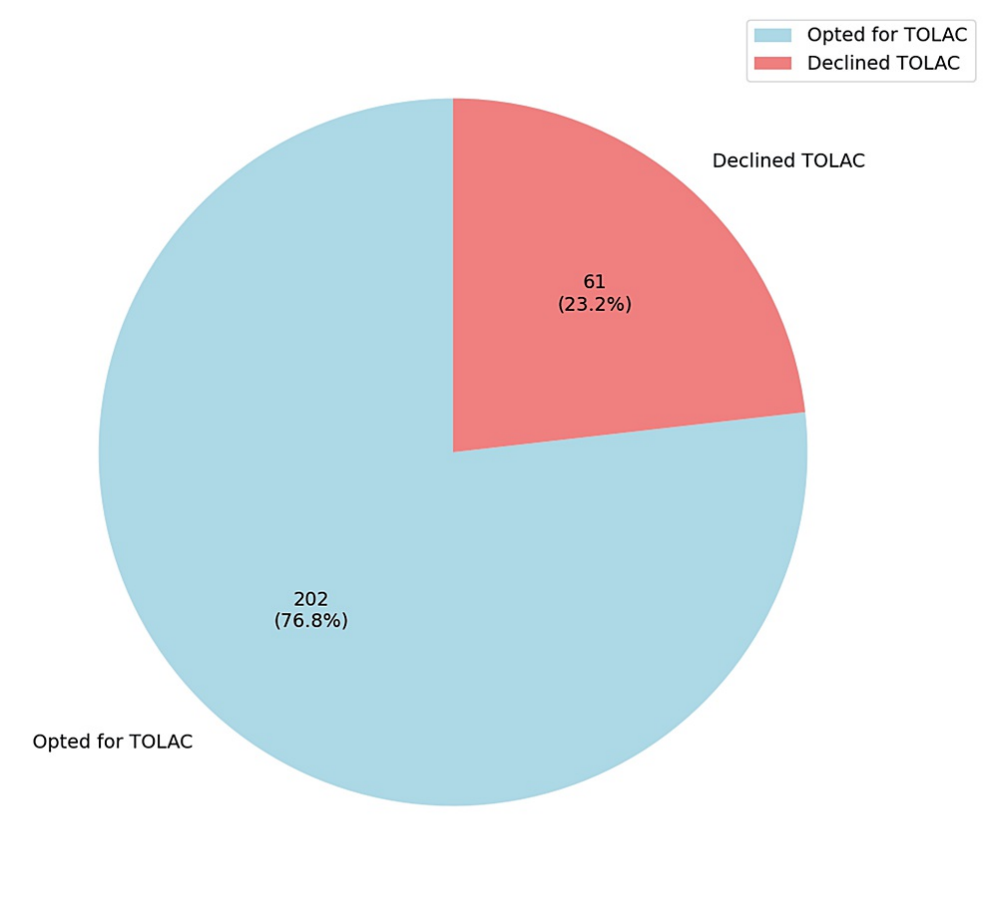
Proportion of women who opted for TOLAC versus those who declined TOLAC: Trial of labor after cesarean

**Figure 2:**
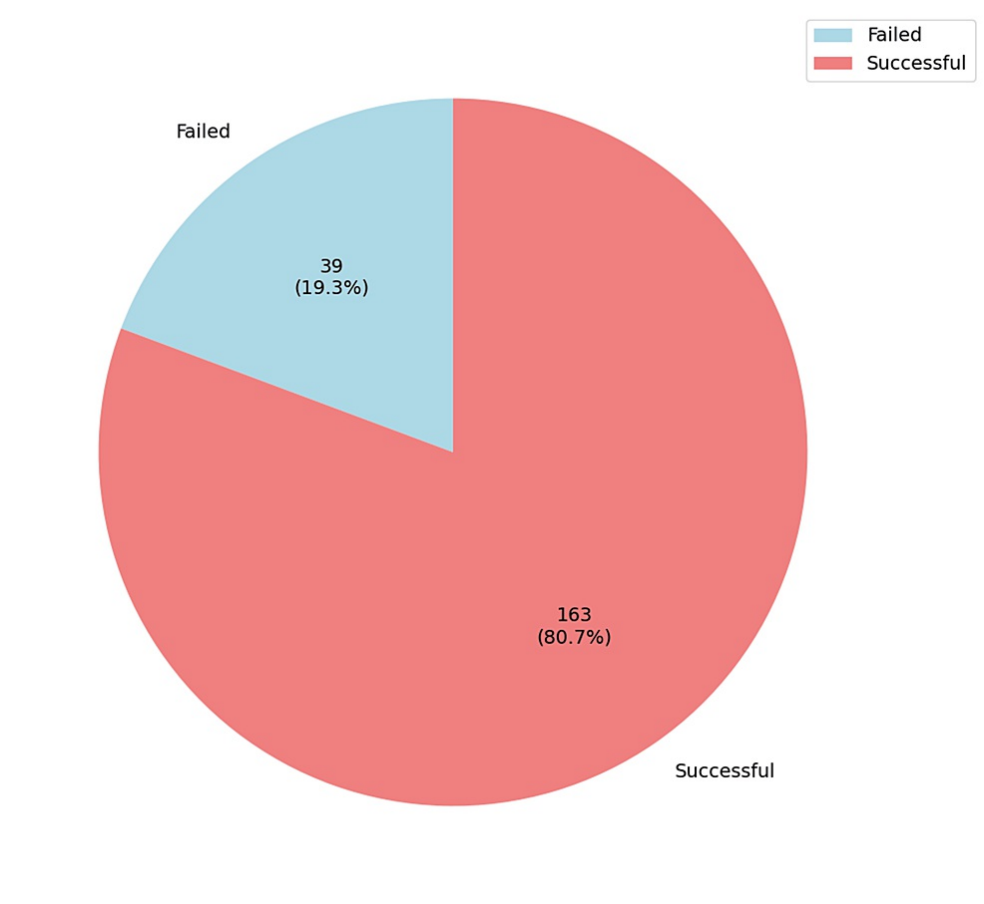
The overall success rate of TOLAC and the number of urgent Cesareans following TOLAC TOLAC: Trial of labor after cesarean

A total of 263 patients were included in this study. Of these, 23.2% (n=61) declined the option of a TOLAC, whereas a substantial 76.8% (n=202) consented to attempt TOLAC (Figure 1). A successful VBAC was achieved in 80.69% (n=163) of the latter group. The remaining 19.31% (n=39) required an emergent cesarean section following TOLAC (i.e., TOLAC failure) (Figure 2).

**Figure 3:**
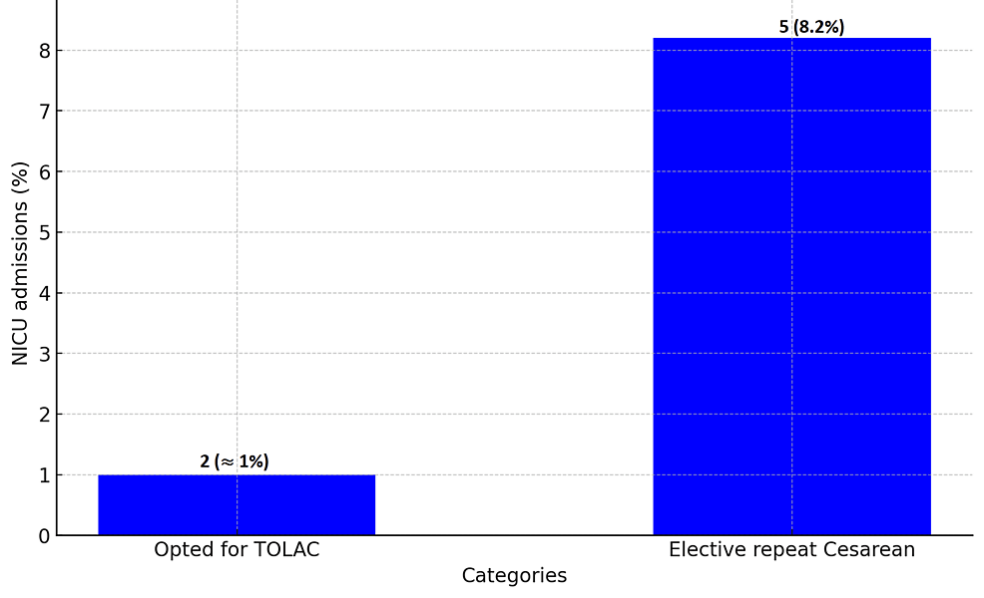
NICU admission rates for TOLAC versus elective repeat cesarean sections TOLAC: Trial of labor after cesarean

**Figure 4:**
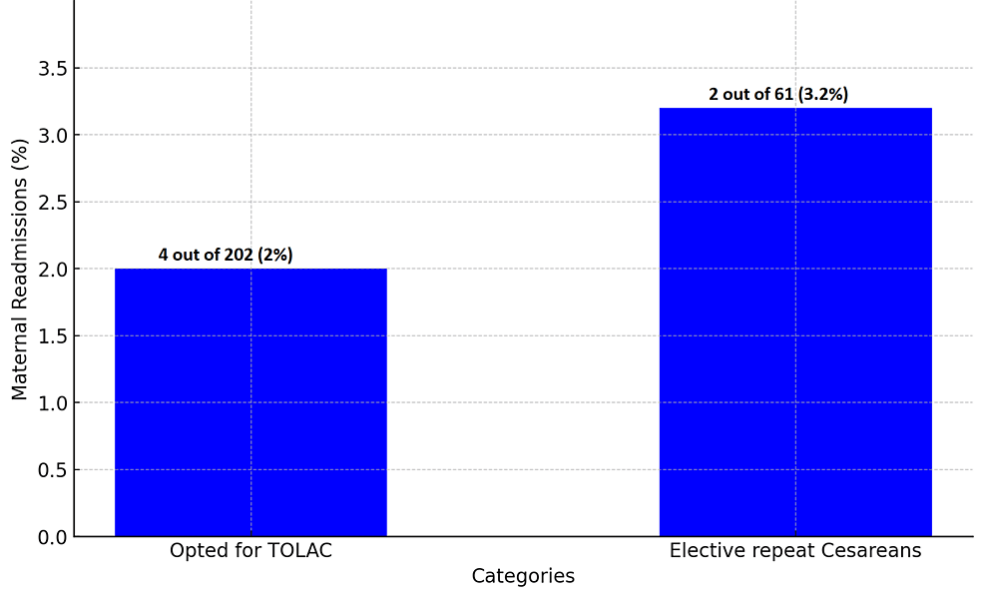
Maternal readmission rates for TOLAC versus elective repeat cesarean sections TOLAC: Trial of labor after cesarean

In the ERCS cohort, 8.2% (n=5) of neonates were admitted to the NICU. This contrasts with less than 1% (n=2) of neonates born to those who pursued TOLAC (Figure 3). Maternal readmission rates were observed at 2% (n=4) for the TOLAC group, of which three cases were unsuccessful VBACs resulting in urgent cesareans, and one case followed a successful VBAC. In comparison, the ERCS group exhibited a maternal readmission rate of 3.2% (n=2) (Figure 4).

**Figure 5:**
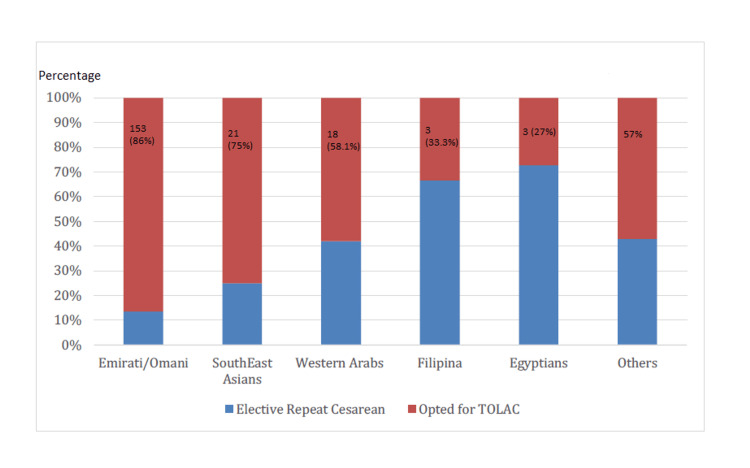
Maternal readmission rates for TOLAC versus elective repeat cesarean sections TOLAC: Trial of labor after cesarean

**Figure 6:**
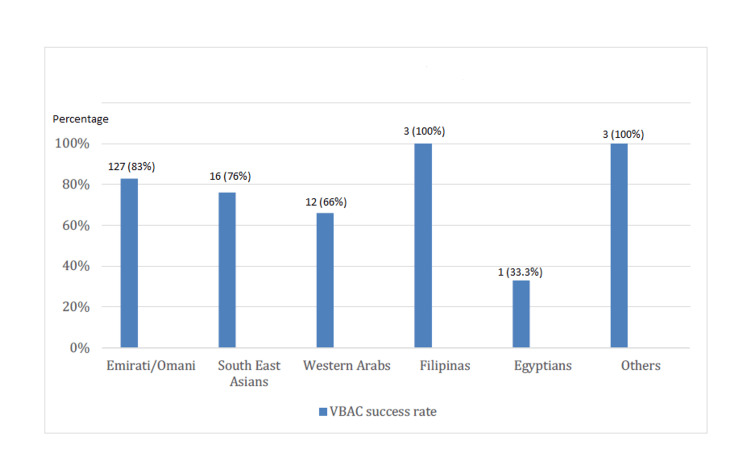
VBAC success rates across different nationalities VBAC: Vaginal birth after cesarean

Breaking down the demographics of the study, 67.3% (n=177) of the participants were Emirati or Omani nationals. A notable 86% (n=153) of this subgroup chose TOLAC, with 83% (n=127) culminating in successful VBACs. Conversely, only 27% (n=3) of the 11 Egyptian participants opted for TOLAC, with a 33.3% (n=1) success rate in VBAC. Among the Western Arabs, a diverse category encompassing women from Syria, Jordan, Palestine, Sudan, Iraq, and others, 51% (n=18) pursued TOLAC, achieving a 66% (n=12) success rate for VBAC. For the Southeast Asians, 75% (n=21 out of 28) elected TOLAC, with a 76% (n=16) success rate. The Filipino subgroup, though smaller in number, had a 100% success rate, with all three participants who chose TOLAC having a successful VBAC (Figures 5, 6).

**Figure 7:**
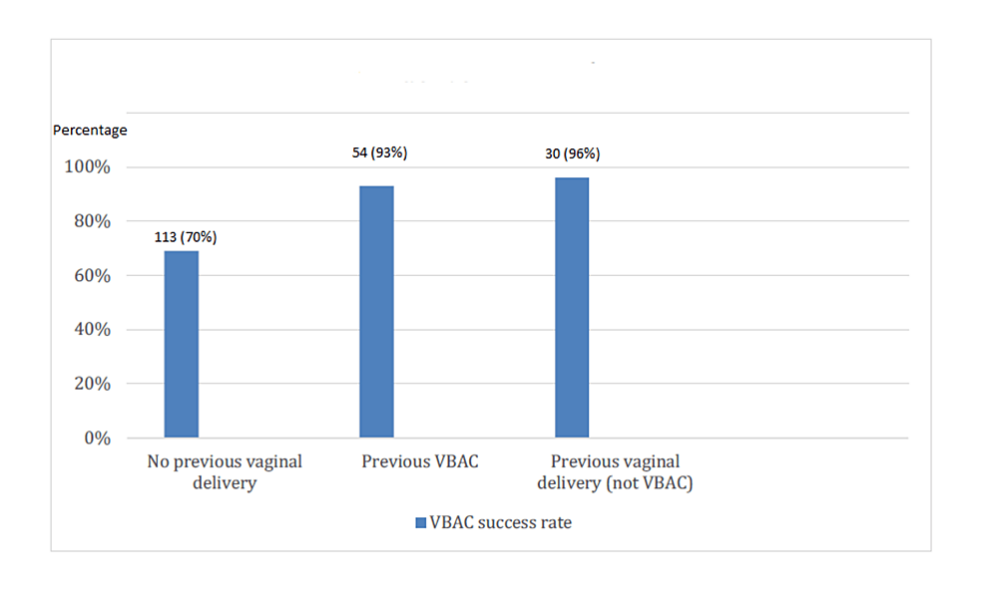
Impact of previous modes of delivery on VBAC success rates VBAC: Vaginal birth after cesarean

No instances of uterine scar dehiscence were reported within the study period. For those patients opting for TOLAC, the subgroup with prior VBACs had a high success rate of 93% (n=54 out of 58), and an even higher rate was seen among those with previous vaginal deliveries not classified as VBAC, at 96% (n=30 out of 31). Patients without any prior vaginal delivery experienced a 70% success rate (n=79 out of 113) (Figure 7).

**Figure 8:**
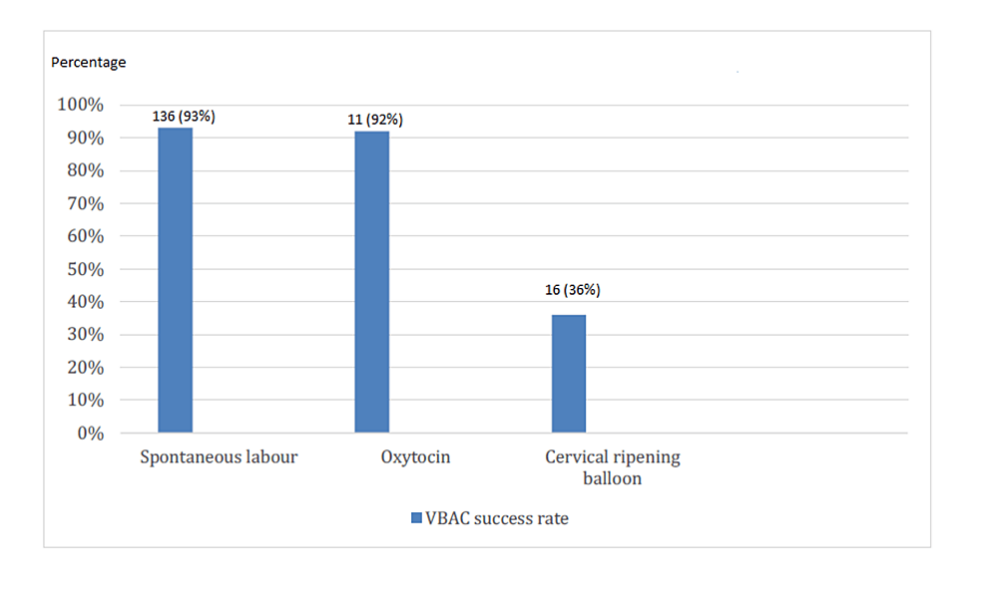
VBAC success rates for different induction methods VBAC: Vaginal birth after cesarean

Among the induced labor subgroup, 12 patients with a favorable cervix received oxytocin and had a 92% success rate for VBAC. In contrast, only 36% (n=16) of the 44 patients with an unfavorable cervix who underwent cervical ripening balloon insertion achieved a successful VBAC (Figure 8).

Statistical analysis was conducted to evaluate the significance of observed differences in VBAC success rates across different nationalities. A chi-square test was performed, assessing the likelihood that these differences could occur by chance. The analysis revealed a chi-square statistic of 7.14 and a p-value of approximately 0.068. This p-value, while not below the conventional threshold of 0.05 for statistical significance, suggests a trend towards significance, indicating that there might be differences in VBAC success rates among different national groups. Specifically, this trend was most evident between the groups of Emirati/Omani women, Egyptian women, Western Arabs, and Southeast Asians. Although not statistically significant at the 0.05 level, these findings hint at the possible influence of cultural factors on VBAC outcomes, warranting further investigation.

## Discussion

Interpretation of key findings

Potential Cultural Influence on VBAC Decisions

While our study did not find a statistically significant difference in VBAC success rates across different national groups (p-value of approximately 0.068), it revealed a notable trend that suggests a potential influence of cultural factors on decisions for TOLAC and VBAC. This observation aligns with the exploratory nature of our analysis and indicates that cultural considerations may play a role in shaping these decisions, albeit not conclusively demonstrated within the statistical significance threshold of our current study. This trend towards a cultural influence observed particularly among the groups of Emirati/Omani women, Egyptian women, Western Arabs, and Southeast Asians, echoes the broader discourse on the importance of cultural context in medical decision-making. Our findings highlight the need for further, more detailed investigations into how specific cultural beliefs and practices might affect the choices and outcomes related to TOLAC and VBAC. This is particularly relevant in the UAE, a culturally diverse nation, emphasizing the role of cultural and ethnic backgrounds in childbirth choices and highlighting the necessity for culturally tailored counseling in obstetric care. Such future studies could help in tailoring more culturally sensitive and informed approaches to counseling and care for women considering TOLAC, potentially improving VBAC success rates and overall satisfaction with the birthing process, as discussed in prior research (McKenzie-McHarg, 2004) [6].

Success Rates and Previous Vaginal Deliveries

The higher success rate of VBAC in patients with prior vaginal deliveries, including previous VBACs, aligns with the study by Landon et al. (2004) [3], which found that a history of vaginal delivery is a strong predictor of successful VBAC. This suggests the need for personalized risk assessments based on obstetric history during TOLAC counseling.

Influence of Induction Methods on VBAC Success

The variation in VBAC success rates depending on induction methods observed in this study provides key insights, in line with the findings of Burris et al. (2012) [7]. They noted that induction of labor, particularly with a favorable cervix, has a significant impact on VBAC outcomes.

Comparison with existing literature

NICU Admissions and Maternal Readmissions

The lower NICU admission rates in the TOLAC group, compared to the ERCS group, are consistent with the findings of Macones et al. (2005) [8], who reported fewer neonatal intensive care admissions in TOLAC cases. This supports the potential neonatal benefits of TOLAC over ERCS. Furthermore, the maternal readmission rates in our study provide a crucial understanding of postpartum complications, echoing the research conducted by Lydon-Rochelle et al. (2001) [9].

Counseling and Decision-Making

The impact of counseling on TOLAC decisions, especially regarding perceptions about pelvic size and previous cesarean narratives, is supported by the work of Blix et al. (2014) [10]. They highlighted the profound impact of perinatal counseling on the decision-making process for TOLAC.

Clinical implications

Tailoring Counseling Based on Cultural Background

Our study underscores the need for culturally sensitive counseling in the UAE, in agreement with the recommendations of Chigbu et al. (2007) [11], who advocate for the consideration of cultural aspects in maternity care to improve patient outcomes.

Risk Communication in VBAC Counseling

The absence of scar dehiscence in our cohort, despite its occurrence in other studies, highlights the importance of balanced risk communication, as emphasized by Taran et al. (2008) [12]. They advocated for comprehensive, evidence-based discussions with patients considering TOLAC.

Expanding the Scope of Cultural Influences on VBAC Decision-Making

The complexities of VBAC decisions are not only shaped by individual choices but also by broader societal norms and healthcare practices. This multifaceted aspect is explored in the study by Chen et al. (2021) [13], which examines the impact of a decision aid for shared decision-making on birth choices after cesarean in Taiwan, highlighting the significant role of informed choice in VBAC. Additionally, Sanchez and McCash (2019) [14] discuss the critical role of nurses in facilitating decision-making for VBAC, emphasizing the importance of support and knowledge provided by healthcare professionals. Furthermore, Chen et al. (2018) [15] delve into the decision-making processes of women in Taiwan and the various influences on their mode of birth following a previous cesarean section, underscoring the interplay between individual, cultural, and systemic factors. These insights are particularly relevant in the UAE, where cultural diversity necessitates healthcare providers to adopt a culturally competent approach in VBAC counseling, ensuring a nuanced understanding of the diverse needs and expectations of women from different backgrounds.

Limitations

Generalizability

The findings are significant for the UAE population but may have limited applicability in different cultural or national contexts. Future research in diverse settings is essential.

Counseling Practices Variability

The impact of counseling on decision-making was primarily anecdotal. Future research should include standardized methods to assess counseling practices.

Future research directions

Longitudinal Studies

Long-term outcomes of VBAC versus ERCS, especially in culturally diverse populations, are still underexplored and require further investigation.

Qualitative Research

Qualitative studies exploring patient and provider perspectives on VBAC decision-making in multicultural settings could further enrich our understanding.

## Conclusions

This article sheds light on the integral role of cultural influences and obstetric history in the decision-making and outcomes of vaginal birth after cesarean (VBAC). The study underscores the variance in VBAC acceptance and success rates among diverse cultural groups, with local Emirati and Omani women showing a higher preference for trial of labor after cesarean (TOLAC). Key findings also include higher VBAC success rates in patients with prior vaginal deliveries and spontaneous labor, alongside lower NICU admissions and maternal readmissions in the TOLAC group. These insights advocate for the importance of personalized, culturally sensitive counseling in the context of VBAC. The study, while presenting valuable contributions to the field, recognizes its limitations in terms of generalizability and the need for further in-depth research in this area.

## References

[1] Osterman MJK, Martin JA (2019). Trends in Low-risk Cesarean Delivery in the United States, 1990-2016. National Vital Statistics Reports,Centers for Disease Control.

[2] American College of Obstetricians and Gynecologists (2019). ACOG Practice Bulletin No. 205: vaginal birth after cesarean delivery. Obstet Gynecol.

[3] Landon MB, Hauth JC, Leveno KJ (2004). Maternal and perinatal outcomes associated with a trial of labor after prior cesarean delivery. N Engl J Med.

[4] Xierali IM, Nivet MA, Rayburn WF (2017). Relocation of obstetrician-gynecologists in the United States, 2005-2015. Obstet Gynecol.

[5] Guise JM, Eden K, Emeis C (2010). Vaginal birth after cesarean: new insights. Evid Rep Technol Assess (Full Rep).

[6] McKenzie-McHarg K (2004). Traumatic birth: understanding predictors, triggers, and counseling process is essential to treatment. Birth.

[7] Burris HH, Rifas-Shiman SL, Kleinman K (2012). Vitamin D deficiency in pregnancy and gestational diabetes mellitus. Am J Obstet Gynecol.

[8] Macones GA, Peipert J, Nelson DB (2005). Maternal complications with vaginal birth after cesarean delivery: a multicenter study. Am J Obstet Gynecol.

[9] Lydon-Rochelle M, Holt VL, Easterling TR, Martin DP (2001). Risk of uterine rupture during labor among women with a prior cesarean delivery. N Engl J Med.

[10] Blix E, Kumle M, Kjærgaard H, Øian P, Lindgren HE (2014). Transfer to hospital in planned home births: a systematic review. BMC Pregnancy Childbirth.

[11] Chigbu CO, Ezeome IV, Iloabachie GC (2007). Cesarean section on request in a developing country. Int J Gynaecol Obstet.

[12] Taran A, Ignatov A, Smith B, Bischoff J, Costa SD (2008). Methotrexate monotherapy for high-risk gestational trophoblastic neoplasia after therapy with etoposide, methotrexate, and dactinomycin: a case report. Am J Obstet Gynecol.

[13] Chen SW, Yang CC, Te JC, Tsai YL, Shorten B, Shorten A (2021). Birth choices after caesarean in Taiwan: a mixed methods pilot study of a decision aid for shared decision making. Midwifery.

[14] Sanchez E, McCash L (2023). Do women have a choice? Nurse’s role in decision making regarding vaginal birth after cesarean. https://sigma.nursingrepository.org/handle/10755/16872.

[15] Chen SW, Hutchinson AM, Nagle C, Bucknall TK (2018). Women's decision-making processes and the influences on their mode of birth following a previous caesarean section in Taiwan: a qualitative study. BMC Pregnancy Childbirth.

